# An Adaptive Orientation Estimation Method for Magnetic and Inertial Sensors in the Presence of Magnetic Disturbances

**DOI:** 10.3390/s17051161

**Published:** 2017-05-19

**Authors:** Bingfei Fan, Qingguo Li, Chao Wang, Tao Liu

**Affiliations:** 1 State Key Laboratory of Fluid Power and Mechatronic Systems, School of Mechanical Engineering, Zhejiang University, Hangzhou 310027, China; bingfeifan@foxmail.com (B.F.); cwangzju@foxmail.com (C.W.); 2 Department of Mechanical and Materials Engineering, Queen’s University, Kingston, ON K7L 3N6, Canada; ql3@queensu.ca

**Keywords:** inertial and magnetic sensors, orientation estimation, magnetic disturbances, instrumented gimbal, gradient descent algorithm

## Abstract

Magnetic and inertial sensors have been widely used to estimate the orientation of human segments due to their low cost, compact size and light weight. However, the accuracy of the estimated orientation is easily affected by external factors, especially when the sensor is used in an environment with magnetic disturbances. In this paper, we propose an adaptive method to improve the accuracy of orientation estimations in the presence of magnetic disturbances. The method is based on existing gradient descent algorithms, and it is performed prior to sensor fusion algorithms. The proposed method includes stationary state detection and magnetic disturbance severity determination. The stationary state detection makes this method immune to magnetic disturbances in stationary state, while the magnetic disturbance severity determination helps to determine the credibility of magnetometer data under dynamic conditions, so as to mitigate the negative effect of the magnetic disturbances. The proposed method was validated through experiments performed on a customized three-axis instrumented gimbal with known orientations. The error of the proposed method and the original gradient descent algorithms were calculated and compared. Experimental results demonstrate that in stationary state, the proposed method is completely immune to magnetic disturbances, and in dynamic conditions, the error caused by magnetic disturbance is reduced by 51.2% compared with original MIMU gradient descent algorithm.

## 1. Introduction

Micro-electromechanical systems (MEMS)-based magnetic and inertial sensors have been widely used for human motion analysis due to their advantages of low cost, light weight and compact size. The applications include walking speed estimation [1], hand pose and kinematics estimation [2,3], knee-joint kinematics [4] and daily-life activity assessment [5]. Generally, a typical magnetic/inertial measurement unit (MIMU) consists of a tri-axial accelerometer, a tri-axial gyroscope and a tri-axial magnetometer. Obtaining sensor orientation is unavoidable for most of the applications and therefore, accurate orientation estimation from the MIMU is critical. Theoretically, the attitude of a sensor can be calculated from the measured gravitational acceleration, and the heading can be calculated from the measured geomagnetic field. Both of them can also be updated by the integration of angular velocity. Each sensor has its own limitations [6]. Accelerometer can only determine the attitude accurately in stationary state or when the movement acceleration is negligible. Gyroscope orientation update will suffer from gyro drift, and magnetometer data is easily distorted by the so-called hard-iron and soft-iron distortions [7].

In order to improve the accuracy of orientation estimation, sensor fusion is always required. A variety of fusion algorithms have been proposed in the literature. Kalman filter represents the most common algorithm, which is a recursive Bayesian state estimation approach [8]. There are many different implementations of Kalman Filters, including Extended Kalman Filter (EKF) for nonlinear models [8], unscented Kalman filter [9], delay Kalman Filter [10] and dual Kalman filter [11]. Kalman filter based algorithms are considered to be highly accurate and effective. Nonlinear complementary filter [12] and gradient descent algorithm [13] are the other popular fusion algorithms, they are also reported achieving good performance. Through these developments, the orientation estimation accuracy has been improved gradually. However, the accuracy is easily influenced by external disturbances, among which the impact of the magnetic disturbances is a long-standing issue [14]. This is due to the fact that the magnetic field is easily affected by ferromagnetic materials in indoor surroundings [15], such as the steel in the floor, machines, computers and mobile phones. The root mean square error (RMSE) of heading error caused by the magnetic disturbances can reach up to 15.4° [16] sometimes. 

To mitigate the negative effect of the magnetic disturbances, several methods [16,17,18,19,20] were proposed to reduce heading error under the presence of magnetic disturbances. A threshold-based method was proposed in [17]. When the measured magnetic field exceeds the threshold, the algorithm would discard the measured magnetic field and replace it with the predicted magnetic field. The major problem is that it is difficult to select a perfect threshold. When the magnetic field is close to the threshold, frequent switching between the measured and predicted magnetic field would make the algorithm unstable. A novel quaternion-based complementary filter was presented in [21]. The filter avoided the impact of the magnetic disturbances on the roll and pitch, but heading is still seriously influenced. Yadav et al. [16] proposed a particle filter-based approach, which performed online detection and compensation for magnetic disturbances, then performed the correction using an adaptive cost function by adapting the variance during particle resampling of the particle filter. The RMSE of orientation estimation is much smaller than the uncompensated. However, this particle filter-based approach is complex and difficult to be a general method which can be directly applied to other algorithms, and the heading would still be influenced in stationary state. Most recently, a magnetic disturbances compensation method based on gradient descent algorithm was proposed in [19]. This method distinguished magnetic disturbances states by setting a magnetic magnitude threshold, and it is indeed an improved version of existing algorithm aiming at magnetic disturbances. However, the threshold determination process is troublesome [22], and the set threshold cannot cope with magnetic disturbances of small magnitude error but with large dip angle error.

By reviewing the related scientific literature, we believe one key area for promoting MIMU’s application in real world applications is to develop adaptive strategies to cope with magnetic disturbances, rather than to invent new ways for fusing sensor measurements. Thus, in this paper, we propose a general magnetic disturbance rejection method which can be easily applied to existing state of art fusion algorithms, either Kalman filter based or gradient descent based fusion algorithms. Here, the gradient descent fusion algorithm is selected as an example of the implementation. The performance of the proposed method is compared with the original gradient descent algorithm on a customized instrumented gimbal.

## 2. Materials and Methods

### 2.1. Sensor Orientation Representation

The sensor orientation can be expressed in several ways including Euler angles, quaternion, axis angle, and rotation matrix, etc. [23]. Among these representations, Euler angles and quaternion are the most common methods. Euler angles represent a sequence of three elemental rotations. Although it may lead to ambiguous results and singularity problems [21], it is easy to visualize and understand. While the quaternion is explicit and do not have singularity problem. Moreover, it is easy for interpolation, the quaternion representation is defined as:

(1) qES=[q0q1q2q3]=[cosθ2exsinθ2eysinθ2ezsinθ2]

where ∥qES∥=1. $e=\left[e_{x}\;e_{y}\;\;e_{z}\right]$ denotes a unit vector representing the rotation axis. θ is the rotation angle.

The sequence of the Euler angles used in this paper is in the ZYX order. The rotations around Z, Y and X are called yaw (ψ), pitch (θ), and roll angle (ϕ), respectively. Yaw angle is also called heading in some systems [24].

### 2.2. Sensor Fusion Algorithm: Gradient Descent Algorithm

In this paper, we adopt the gradient descent algorithm [13] as the basic sensor fusion algorithm. The gradient descent algorithm is an approach used to find the minimum of a function [25], which has been widely used with inertial sensors [13,14,19,24]. The objective function is defined as Equations (2) and (3). The objective of the orientation estimation is to find the optimized quaternion that minimizes the objective function, which can be interpreted as to minimize the error between the measured field and the reference field:

(2) minq^ES ∈ℜ4f(q^ES,d^E,s^S )

(3) f(q^ES, d^E,s^S)=q^ES*⊗ d^E⊗ q^ES−s^S

where q^ES is the estimated orientation and q^ES* is its conjugate quaternion. d^E denotes a predefined reference field in the earth frame, such as gravity acceleration and geomagnetic field. s^S is the measured field in the sensor frame.

The gradient of the cost function ∇f is defined as Equation (4). J is its Jacobian:

(4) ∇f(q^ESk, d^E,s^S)=JT(q^ESk, d^E)f(q^ESk, d^E,s^S)

For the accelerometer estimation, substitute gravity vector g^E=[0 0 0 1] for d^E and the accelerometer data a^S=[0 ax ay az] for s^S, and for magnetometer estimation, substitute the normalized magnetic vector b^E=[0 bx 0 bz] for d^E and the magnetometer data m^S=[0 mx my mz] for s^S.

The gradient descent algorithm can be applied to both inertial measurement unit (IMU) and MIMU. The IMU algorithm fuses data from accelerometers and gyroscopes, while the MIMU algorithm fuses data from accelerometers, gyroscopes and magnetometers. The ∇f of the IMU and MIMU algorithms are defined as Equation (5):

(5) ∇f={JgT(q^ES )fg(q^ES,a^S ) for IMU Jg,bT(q^ES, b^E)fg,b(q^ES,a^S, b^E,m^S) for MIMU 

and for the MIMU, its cost function and Jacobian are defined as Equations (6) and (7), respectively:

(6) fg,b(q^ES,a^S, b^E,m^S)=[fg(q^ES,a^S )fb(q^ES, b^E,m^S)]

(7) Jg,bT(q^ES, b^E)=[JgT(q^ES )JbT(q^ES, b^E)]

By applying gradient descent algorithm, the final derived mathematical model of the gradient descent algorithm is described in Equations (8) and (9):

(8) qESt=q^ESt−1+q˙EStΔt

(9) q˙ESt=q˙ESω,t−β∇f||∇f||

where qESt represents the orientation of the earth frame relative to sensor frame at time *t*.  q˙ESω,t is the change rate of the orientation calculated from angular velocity integral. q˙ESt denotes the quaternion derivative describing the compensated change rate of the orientation. ∇f is the gradient of the objective function defined in Equation (4). Δt represents the sampling period, and β is the tuning parameter related to gyroscope. 

In the gradient descent algorithm, there is only one adjustable parameter β (a gyroscope measurement error related parameter), which is described as follows:

(10) $$\beta =∥\frac{1}{2}\hat{q}⊗\left[0\;{\tilde{\omega }}_{max}\;{\tilde{\omega }}_{max}\;{\tilde{\omega }}_{max}\right]∥=\sqrt{\frac{3}{4}}{\tilde{\omega }}_{max}$$

where ${\tilde{\omega }}_{max}$ denotes the maximum gyroscope measurement error of each axis, and $\hat{q}$ is an arbitrary unit quaternion. 

The gradient descent algorithm is easy to implement and its performance is reported equivalent to Kalman filter based algorithms [13]. The IMU algorithm is immune to magnetic disturbances, but the yaw angle is an angle relative to the initial state rather than the absolute angle, and it would suffer from drift error because of the lack of a reference vector in the transverse plane. In contrast, the MIMU algorithm offers the absolute yaw angle and the drift error can be compensated by the measured magnetic field. However, the yaw angle is easily influenced by the magnetic disturbances. Taking all these factors into account, a better fusion algorithm should take advantages of both the IMU and MIMU fusion algorithms.

### 2.3. The Proposed Adaptive Method

**Figure 1:**
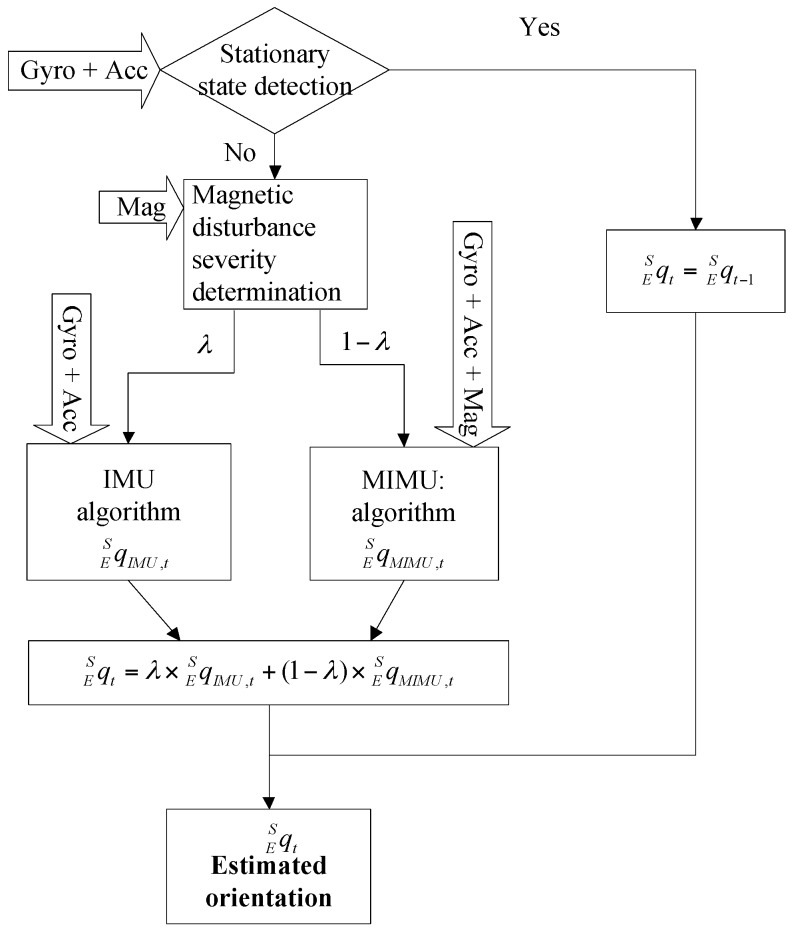
Overview of the proposed method’s structure.

The proposed method is a combination of the IMU algorithm and the MIMU algorithm. Overview of the proposed method’s structure is shown in Figure 1.

In the proposed method, the sensor data is preprocessed to distinguish special states, including stationary state and magnetic distortion state. Different strategies are developed for different states, so as to improve the accuracy of orientation estimation. The preprocessing contains two steps: stationary state detection and magnetic disturbance severity determination. The measured angular velocities and accelerations are used to determine the stationary state, and the magnetic field is used to determine the severity of the magnetic disturbances. The details of the state determination methods are described in the following.

#### 2.3.1. Stationary State Detection

For the MIMU algorithm, when the sensor is in a stationary state, the yaw angle will finally converge to the direction of measured magnetic field [20]. If the surrounding magnetic field were a distorted magnetic field, large errors in yaw angle would be induced unavoidably. However, if the stationary state were detected, the best way is to keep the estimated orientation unchanged. So that the estimated orientation can be immune to any kind of magnetic disturbances. Thus, the critical issue is to detect the stationary state. Fortunately, the accelerometer and gyroscope are sensitive to motion. Ideally, when the sensor is in stationary state, the angular velocity should be zero and the acceleration of each axis should remain unchanged. These features can be adopted as a criterion for the stationary state detection [26]. Thresholds can be set for accelerometer and gyroscope in order to detect the stationary state. The accelerometer and gyroscope stationary state criterions are depicted in Equations (11) and (13).

1. Accelerometer stationary state detection:

(11) $$\{\begin{array}{c} \left|a_{x_{t}}-a_{x_{t-t_{0}}}\right|<th_{a}\\ \left|a_{y_{t}}-a_{y_{t-t_{0}}}\right|<th_{a}\\ \left|a_{z_{t}}-a_{z_{t-t_{0}}}\right|<th_{a} \end{array}$$

where $a_{x_{t}}$ denotes the acceleration measured along X axis at time t. $t_{0}$ is an interval. $a_{x_{t-t_{0}}}$ denotes the acceleration measured at time $t-t_{0}$. $th_{a}$ represents the stationary state threshold. Other notations for Y and Z axes are similar to X axis.

**Figure 2:**
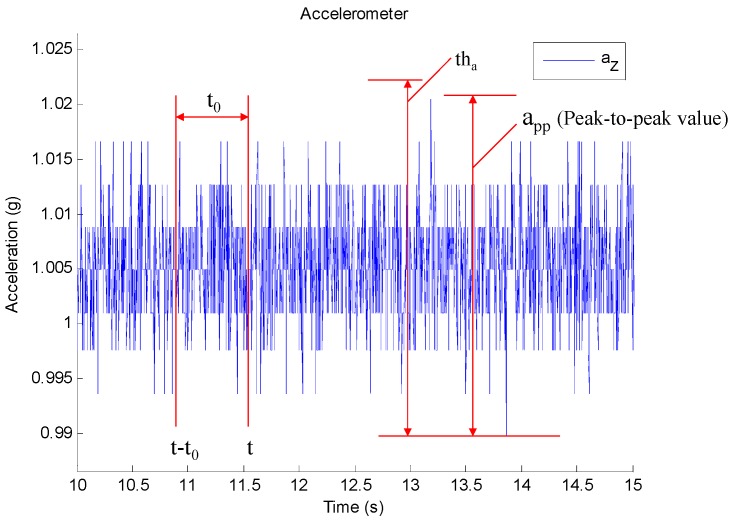
An example of collected z-axis acceleration in stationary state.

The stationary state criterion can be interpreted as that the variation of the measured acceleration along each axis is small than $th_{a}$ in an interval $t_{0}$, which ensures the sensor’s orientation is unchanged in the period of $t_{0}$. Figure 2 shows an example of collected z-axis acceleration in stationary state. $t_{0}$ determines the latency between the actual and the detected stationary state [27]. A long interval make the method insensitive to stationary state and a short interval may lead to frequent switching between dynamic and stationary state. The optimum $t_{0}$ should be determined by stationary detect test. $th_{a}$ can be determined according to accelerometer noise in theory, but the noise varies from sensor to senor, so it is more appropriate to choose $th_{a}$ by tuning experiments. As Figure 2 shows, $th_{a}$ can be calculated using Equation (12):

(12) $$th_{a}=k\;a_{pp},\;k=1.2\sim 1.5$$

where $a_{pp}$ is the peak to peak value when the sensor is in stationary state. k is a tolerance factor.

We judge the stationary state by individual accelerations $a_{x}$ $a_{y}$ $a_{z}$ rather than the resultant acceleration [20], because slow rotation cannot be detected by the resultant acceleration.

2. Gyroscope stationary state detection:

(13) $$\{\begin{array}{c} |\omega_{x}|<th_{gyro}\\ |\omega_{y}|<th_{gyro}\\ |\omega_{z}|<th_{gyro} \end{array}$$

where $\omega_{x}$, $\omega_{y}$ and $\omega_{z}$ denote the angular velocity measured by the gyroscope. $th_{gyro}$ is the angular velocity threshold for determining whether the sensor is in stationary state.

The $th_{gyro}$ can be set according to the noise level of gyroscope. The selected $th_{gyro}$ should be sensitive to motion, so as to determine the state correctly. The stationary state condition for gyroscope can be interpreted as that the angular velocity around each axis should be smaller than the threshold. Gyroscope stationary state detection is used as a supplement to acceleration stationary state detection because rotation around Z axis cannot be detected by accelerometer. Therefore, the detected stationary state should meet both conditions described in Equations (11) and (13). 

#### 2.3.2. Magnetic Disturbance Severity Determination

The proposed method can be regarded as an implementation of the first order complementary filter, which is based on the complementary properties of the IMU algorithm and MIMU algorithm. The model of the proposed method is given by Equation (14):

(14) qESt=λqMIMU,tES+(1−λ)qMIMU,tES 0≤λ≤1 

where qESt denotes the orientation of the earth frame relative to sensor frame at time *t*. qIMU,tES and qMIMU,tES are the orientations estimated by IMU fusion algorithm and MIMU fusion algorithm respectively. λ and (1−λ) are the weights applied to each orientation estimation.

**Figure 3:**
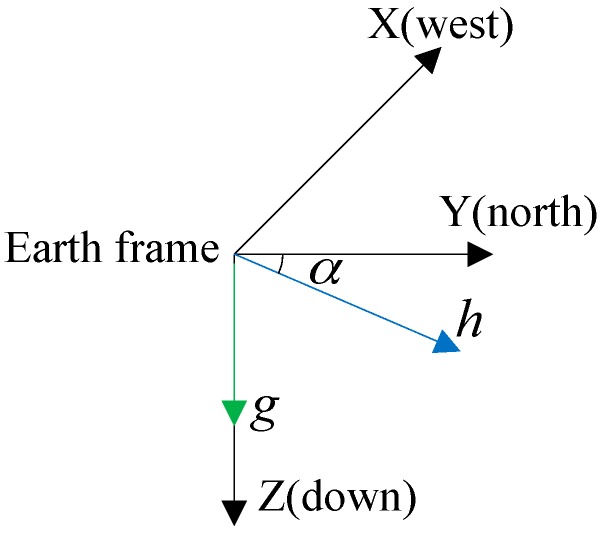
The geomagnetic field in the Earth frame. g is the gravity acceleration. *h* is the geomagnetic field. The length of *h* represents the magnitude. The angle α between the horizontal and magnetic field is defined as the dip angle.

λ is regarded as an indicator of magnetic disturbances. When the magnetic disturbance is severe, the final orientation should trust more on $q_{IMU}$ and less on $q_{MIMU}$. Thus, the critical issue is to determine λ according to the current measured magnetic field. Generally, in a magnetic disturbances free environment, the Earth magnetic field should be a constant vector, where both the magnitude and dip angle are constants. Figure 3 shows the gravitational acceleration and geomagnetic field in the Earth frame.

When the magnitude or the dip angle of the measured magnetic field is not stable, it indicates that magnetic disturbances exist in the surroundings [16,17]. There is no clear line between magnetic disturbances free and distorted state, but the disturbance severity can be represented by the deviation percentage. According to this principle, the magnetic disturbance weight λ can be calculated by Equations (15)–(17):

(15) $$\lambda_{1}=\left|\mathrm{norm}\left(\mathrm{mag}\right)-m_{0}\right|/m_{0}\;if\;\lambda_{1}>1,\;\lambda_{1}=1$$

(16) $$\lambda_{2}=\left|\theta_{dip}-\theta_{0}\right|/th_{dip}\;if\;\lambda_{2}>1,\;\lambda_{2}=1$$

(17) $$\lambda =\left(\lambda_{1}+\lambda_{2}\right)/2$$

where $\lambda_{1}$ is the disturbance weight calculated from the magnitude of magnetic field. $\lambda_{2}$ is the weight calculated from dip angle. λ is the final disturbance weight. $m_{0}$ and $\theta_{0}$ denote the magnitude and dip angle of geomagnetic field. They are predetermined during the process of magnetometer calibration under magnetic disturbance free environment. The divisors in Equations (15) and (16) are regarded as the maximum tolerances of the errors. 

The dip angle is calculated by Equation (18):

(18) $$\theta_{dip}=\frac{\pi }{2}-\arccos\left(\left(A\left(q\right)h\right)\cdot g/∥h∥\right)$$

where A(q) is the rotation matrix converted from the estimated orientation of sensor. g is the gravity acceleration, and h is the measured magnetic field.

The properties of indicators $\lambda_{1}$ and $\lambda_{2}$ are complementary, because $\lambda_{1}$ cannot indicate the distorted magnetic field of the same magnitude but with large dip angle error, and $\lambda_{2}$ cannot indicate the distorted magnetic field of the same dip angle but with large magnitude error. Hence, the magnetic disturbances could be indicated more accurately by taking the mean of $\lambda_{1}$ and $\lambda_{2}$ as the final disturbance weight. In addition, the magnetic disturbance weight λ is continuously variable with external magnetic disturbances, which ensures the orientation updated smoothly without abrupt changes.

### 2.4. Accuracy Evaluation Based on Quaternion

The quaternion’s operation is simple and of low computational cost, and comparison analysis based on quaternion is insensitive to Euler-angle’s singularity problems when the second Euler rotation is close to ±90° [28]. Therefore, when singularity problems happens, error analysis should be performed using quaternion comparison instead of Euler angles comparison. Quaternion multiplication and conjugation are the basic operations used in quaternion comparison. Let ⊗ denotes quaternion multiplication, and ${q_{B}}^{*}=\left[q_{0},-q_{1},-q_{2},-q_{3}\right]$ denotes the conjugate quaternion of $q_{B}$. $q_{c}=q_{A}⊗q_{B}$ represents a vector that rotates at $q_{B}$ first, and then rotates at $q_{A}$. $q_{c}={q_{B}}^{*}$ represents a vector that rotates around the axis indicated by $q_{B}$ but in the opposite rotation. Therefore, the error $q_{e}$ between $q_{A}{\;\mathrm{and}\;q}_{B}$ can be described as Equation (19). As a result, the derived $q_{e}$ satisfies Equation (20), which means the orientation represented by $q_{e}$ is relative to $q_{B}$:

(19) $$q_{e}=q_{A}⊗{q_{B}}^{*}$$

(20) $$q_{A}=q_{e}⊗q_{B}$$

$q_{e}$ is also a quaternion which is not intuitive. Converting $q_{e}$ to Euler angles makes it easy to be understood and compared. The conversion is described in Equation (21). Another performance assessment method presented in [8] is also used in this paper, in which the orientation error Δθ is introduced. It is a single indicator that is easy to quantify the improvement of the performance. Δθ is calculated from the scale part of a quaternion as described in Equation (22):

(21) $$\{\begin{array}{c} \phi =\mathrm{atan}2\left(2q_{2}q_{3}+2q_{0}q_{1},\;{q_{3}}^{2}-{q_{2}}^{2}-{q_{1}}^{2}+{q_{0}}^{2}\right)\\ \theta =-\mathrm{asin}\left(2q_{1}q_{3}-2q_{0}q_{2}\right)\\ \psi =\mathrm{atan}2\left(2q_{1}q_{2}+2q_{0}q_{3},\;{q_{1}}^{2}+{q_{0}}^{2}-{q_{3}}^{2}-{q_{2}}^{2}\right) \end{array}$$

(22) $$\Delta \theta =2\;\arccos\left(q_{0}\right)$$

where ψ,θ,φ denote the rotation angles around Z, Y, and X axis, and $q_{0}–q_{3}$ are the components of a quaternion. Δθ is the orientation error. Hence, the estimation error of the proposed adaptive method and the original gradient descent algorithms can be compared and analyzed.

### 2.5. Testing Apparatus

Testing platform was established for validating the performance of the proposed method. The platform contains a MIMU and an instrumented gimbal, and the MIMU is used to collect the acceleration, angular velocity and magnetic field. The orientation output by the instrumented gimbal is regarded as the ground-truth orientation. The details of the apparatus are described as following.

#### 2.5.1. Magnetic/Inertial Measurement Unit

In this study, we selected a commercially available MIMU (x-IMU, x-io Technologies, Bristol, UK) to collect the original inertial data and magnetic data. The x-IMU contains a 3-axis gyroscope, a 3-axis accelerometer and a 3-axis magnetometer, and all the sensors have been calibrated before they are delivered [29]. The collected sensor data can be stored on SD card or transmitted to a computer through USB or Bluetooth. 

#### 2.5.2. Customized Instrumented Gimbal

**Figure 4:**
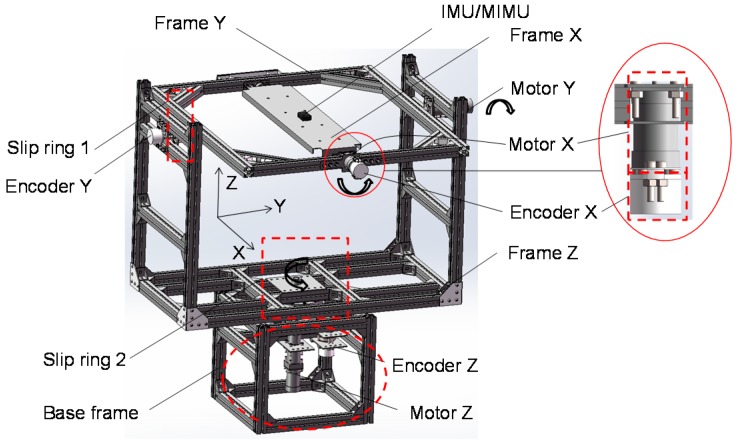
The model of three-axis instrumented gimbal.

In order to evaluate the accuracy of different fusion algorithms, we designed an instrumented gimbal to perform this task. Compared with the traditional stereophotogrammetry, multi-axis gimbal provides a simple and controllable evaluation method by providing known orientations during dynamic movement. More and more literature reported to use the gimbal to benchmark the inertial sensors [30,31]. The gimbal used in our study contains three degrees of freedom (DOFs), corresponding with the three axes of Euler angles. As shown in Figure 4, the gimbal consists of a base frame and three rotatable frames: frame X, Y and Z. Each axis is equipped with a motor and an absolute encoder. The motor makes the frame can be controlled independently. The encoder is capable of outputting the angle around each axis at a resolution of 0.09°. To make each axis rotate continuously, both the power cable and the signal cable are pass through slip rings to avoid cable wrapping problem. Moreover, the gimbal is equipped with a remote control, so that users can control the gimbal wirelessly.

In addition, the testing area of the gimbal should be free of magnetic disturbances. To achieve this feature, the frames of the gimbal are made of aluminum alloy, and the testing area is kept at least 40 cm [32] away from the magnetic disturbances sources such as DC motor, wire, steel bolts. The impact of these ferromagnetic material was verified experimentally, and it was shown that there was no significant difference in magnetometers’ noise level between the testing area and an open area 2 m away from any ferromagnetic materials.

The gimbal’s rotation axis Z, Y and X are consistent with those of the Euler angles, thus these angles and the Euler angles are one-to-one correspondence. The only difference is that the range of the encoders is 0°–360° which is different from that of Euler angles. Consequently, the raw angles of the encoders are first converted to ZYX Euler angles, and then converted to quaternion by using Equation (23) [23], so that the quaternion estimated by fusion algorithms can be compared with that of the gimbal:

(23) $$q=\left[\begin{array}{c} q_{0}\\ q_{1}\\ q_{2}\\ q_{3} \end{array}\right]=\left[\begin{array}{c} \cos(\frac{\varphi }{2})\cos(\frac{\theta }{2})\cos(\frac{\psi }{2})+\sin(\frac{\varphi }{2})\sin(\frac{\theta }{2})\sin(\frac{\psi }{2})\\ \sin(\frac{\varphi }{2})\cos(\frac{\theta }{2})\cos(\frac{\psi }{2})-\cos(\frac{\varphi }{2})\sin(\frac{\theta }{2})\sin(\frac{\psi }{2})\\ \cos(\frac{\varphi }{2})\sin(\frac{\theta }{2})\cos(\frac{\psi }{2})+\sin(\frac{\varphi }{2})\cos(\frac{\theta }{2})\sin(\frac{\psi }{2})\\ \cos(\frac{\varphi }{2})\cos(\frac{\theta }{2})\sin(\frac{\psi }{2})-\sin(\frac{\varphi }{2})\sin(\frac{\theta }{2})\cos(\frac{\psi }{2}) \end{array}\right]$$

where ψ, θ, φ denote the rotation angles around Z, Y, and X axis, and *q* is the derived quaternion. $q_{0}$–$q_{3}$ are the components of the quaternion.

The quaternion derived from the instrumented gimbal is used as a gold standard for evaluating the performance of the proposed method, and the following experiments were carried out on this apparatus.

#### 2.5.3. Sensor Configuration

**Figure 5:**
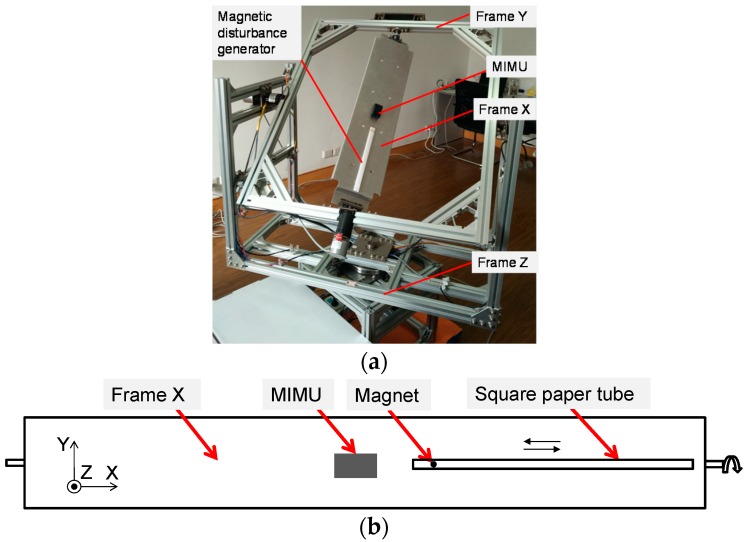
(**a**) MIMU accuracy evaluation setup; (**b**) The detail of the magnetic disturbance generator.

During the experiments, the MIMU was mounted on the testing area of frame X with double-sided tapes, and the X axis of the sensor was aligned with that of the gimbal (Figure 5a). A magnetic disturbance generator was installed beside the MIMU, which is used to simulate the magnetic disturbances in the surroundings. As shown in Figure 5b, the generator consists of a square paper tube and a circular permanent magnet (diameter: 15 mm, thickness: 2 mm). The tube was attached on frame X and the magnet was put into the tube. When frame Y rotated, the magnet would reciprocate in the paper tube due to the gravity. Such that the magnetic disturbance changed with the distance between the magnet and the MIMU.

During the tests, the MIMU’s sensor data were sent to computer for storage through Bluetooth. All the three axes angles of the instrumented gimbal were stored to SD card first, and then converted to quaternion. The sampling rate of the gimbal was 200 Hz and the MIMU was 256 Hz. For comparison, the quaternion from MIMU was downsampled to 200 Hz. The two systems were time-synchronized manually, and all the comparisons were performed using Matlab (The MathWorks Inc., Natick, MA, USA).

### 2.6. Parameters Determination for the Proposed Method

Before the experiments, the parameters described in Equations (11), (13), (15) and (16) should be determined. $m_{0}$, $\theta_{0}$ were determined through the calibration of the magnetometer under magnetic disturbances free condition. The magnetometer of the selected MIMU was calibrated using its accompanying tools x-IMU GUI 13.1 [29]. The calibration process consists of three steps. First, rotate the three frames and collect hard-iron calibration dataset. The rotation speed of Z, Y, X axis of the gimbal were about 40°/s, 70°/s, 360°/s respectively, and the duration was about 30 s. Second, run hard-iron calibration algorithm to calculate the parameters and apply them to the sensor. Finally, collect the magnetic field data in the same way as step one and check the calibration results. 

**Figure 6:**
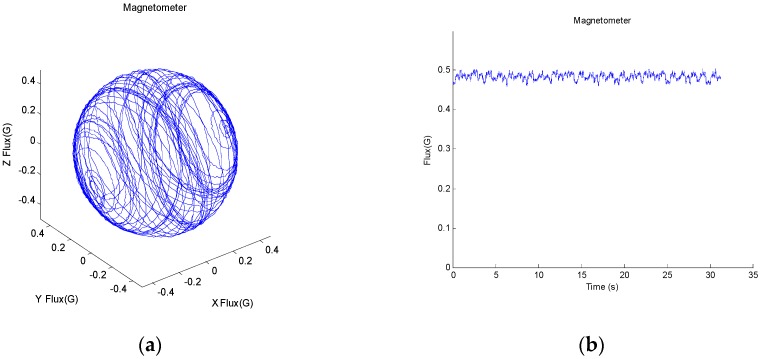
The magnetic field measured by a well calibrated MIMU. (**a**) The measured 3D magnetic field; (**b**) the magnitude of the magnetic field.

After calibration, the output of the magnetometer is shown in Figure 6. It can be seen that the magnitude is nearly a constant, which indicates the sensor has been well calibrated, and the calculated parameters were $m_{0}$ = 0.46 Gs, $\theta_{0}=49.6{}^{\circ}$. The threshold $th_{dip}$ was set as 20° because when the error of the dip angle exceed 20°, it indicates the measured magnetic field is seriously distorted and of little credibility. The method of selecting $t_{0}$, $th_{a}$ and $th_{gyro}$ is similar to that described in [27]. For the accelerometer, values $t_{0\;}$= 0.5 s and $th_{a}=0.04\;g$ were found sufficient for stationary detection. And for gyroscope, the $th_{gyro}$ was set as 0.5°/s and β equals to 0.1 rad/s according to the characteristic of the selected gyroscope. These parameters were applied to the proposed method in the following experiments.

## 3. Experimental Method

The proposed adaptive method was tested under different conditions, including (1) stationary state with magnetic disturbance, (2) dynamic state without magnetic disturbance and (3) dynamic state with magnetic disturbance. Conditions (1) and (2) are used to validate the effectiveness of the stationary state detection, and condition (3) is to test the improvement of the proposed method in dynamic condition. 

### 3.1. Stationary State with Magnetic Disturbance Experimental Protocol

**Figure 7:**
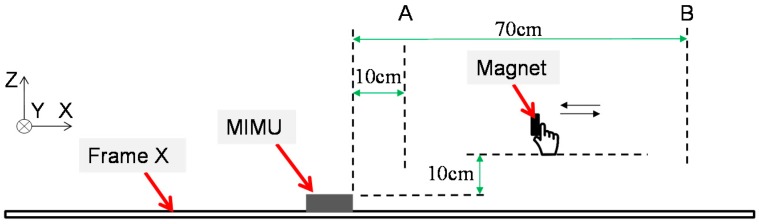
The diagram of stationary state test. The magnet was held over the MIMU, and moved around between boundary A and B.

In this test, as shown in Figure 7, the MIMU was kept in stationary state on the test area of the frame X. In order to simulate the magnetic disturbances in the surroundings, a circular permanent magnet (diameter: 15 mm, thickness: 2 mm) was held over the MIMU and moved near and far away from the MIMU. The distance between the magnet and the MIMU was from 10 cm to 70 cm. The duration of each trial was about 110 s and each trial was repeated for five times. The collected data was used as the input of the original MIMU algorithm and the proposed adaptive method. The Euler angles estimated by the two algorithms were then compared.

### 3.2. Dynamic State without Magnetic Disturbance Experimental Protocol

The stationary state detection of the proposed method should not degrade the performance when the MIMU is not in stationary state. This test was designed to validate this. In this experiment, the MIMU was kept on the gimbal, and the magnet was removed from the magnetic disturbance generator. When performed the experiment, the frames of the gimbal were rotated intermittently at a low speed manually. Thus the motion of the MIMU contained both stationary state and dynamic state. The experiment was repeated for ten times, and each trial lasted about 60 s–80 s. After the experiments, taking the orientation estimated by the original MIMU algorithm as the reference value, the RMSEs of the orientation estimated by the proposed method were calculated and analyzed. The mean of the RMSEs of all the trials were also calculated for statistical analysis.

### 3.3. Dynamic State with Magnetic Disturbance Experimental Protocol

In this experiment, the same magnet mentioned above was mounted on the disturbance generator, so as to generate the magnetic disturbance. To keep each trial consistent, the gimbal was kept at a fixed orientation at the start of the experiment, then start the gimbal by the remote control. All the frames rotated simultaneously and the magnet slid in the paper tube. The rotation speed of axis Z, Y and X were about 40°/s, 70°/s and 360°/s respectively, The duration of each trial was about 60 s and each trial was repeated for ten times. The collected data was used to estimate the orientation by original IMU fusion algorithm, MIMU fusion algorithm and the proposed method. Error analysis was performed based on quaternion comparison as described in Section 2.4. The quaternion error between the gold standard (The instrumented gimbal) and the three methods was converted to Euler angles and orientation error using Equations (21) and (22), and the RMSEs of the Euler angles were compared and analyzed. The mean and standard deviation of all the RMSEs were also calculated for statistical analysis.

## 4. Results

### 4.1. Results under Stationary State with Magnetic Disturbance

**Figure 8:**
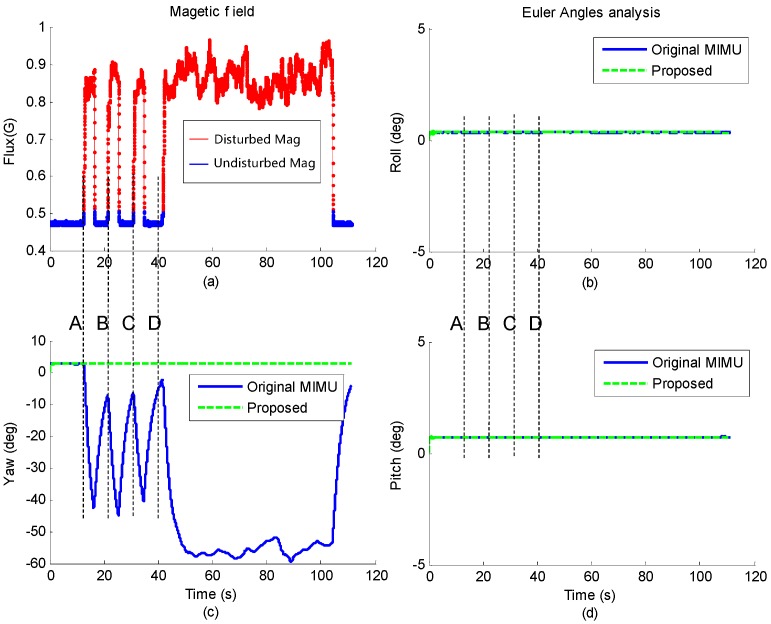
Comparison of Euler angles estimated by different fusion algorithms under stationary state. (**a**) Three-axis magnetic field measured by the magnetometer; The roll (**b**), pitch (**c**) and yaw (**d**) angle estimated by original MIMU gradient descent algorithm and the proposed method.

The magnetometer data in one of the trials is shown in Figure 8a. At time A, B and C, the magnet was moved close to the sensor and then removed after a few seconds, while at time D, the magnet was also moved close to the sensor but stayed for about one minute. It can be seen that as the permanent magnet got close to the MIMU, strong magnetic disturbance was detected by the magnetometer. The corresponding estimated Euler angles of the proposed method and the original MIMU fusion algorithm are shown in Figure 8b–d. 

It also can be seen that the roll and pitch of both methods are not affected after a short time convergence. However, when the magnetic disturbance occurs, the yaw angle of the original MIMU algorithm deviates from the original value dramatically, and the maximum deviation reaches up to 50°. When the disturbance disappears, yaw angle gradually converges to the original value. While for the yaw angle of the proposed method, it is nearly unchanged even when the magnetic disturbance is existing for more than 60 s. Results of the other four trials are similar to this, which demonstrate that the orientation estimated by the proposed method is consistent with the actual situation.

### 4.2. Results under Dynamic State without Magnetic Disturbance

**Figure 9:**
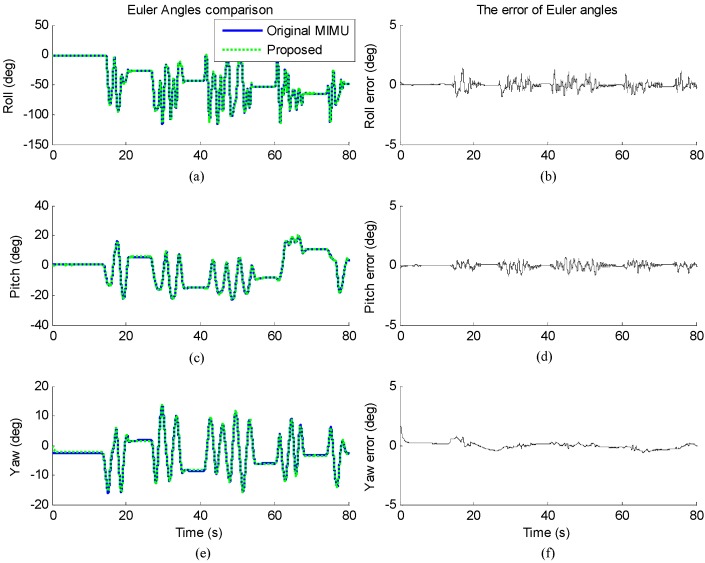
The orientation estimated by original MIMU algorithm (solid) and proposed method (dotted) in dynamic state. (**a**,**c**,**e**) Euler angles; (**b**,**d**,**f**) The error of Euler angles.

Figure 9 shows the results of one of the trials. The Euler angles are estimated by the proposed method and the MIMU algorithm. The mean of RMSEs of roll, pitch and yaw of all the ten trials are 0.43°, 0.17° and 0.39° respectively, which demonstrate that the stationary state detection has little impact on the results of the proposed method in dynamic state, thus will not degrade the performance.

### 4.3. Results under Dynamic State with Magnetic Disturbance

**Figure 10:**
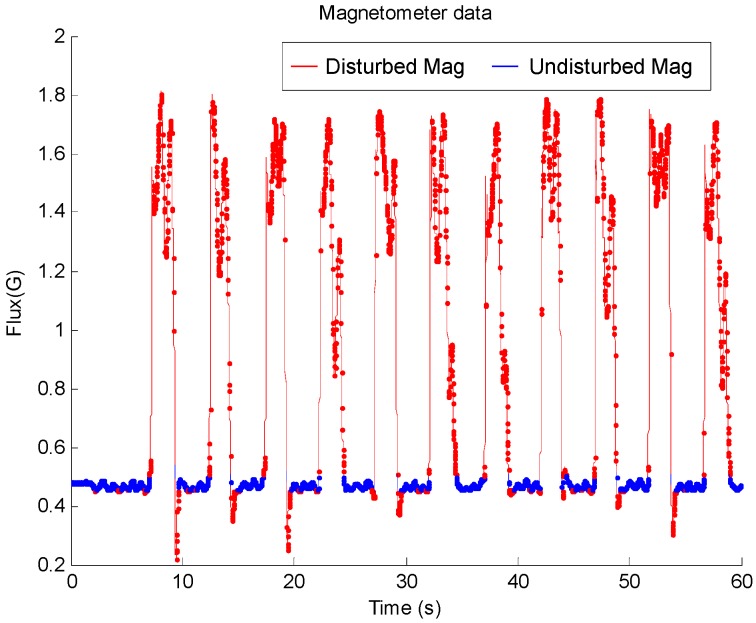
The magnitude of the measured magnetic field during dynamic test.

In one of the trails, the magnitude of the measured magnetic field is shown in Figure 10. The disturbed magnetic field is plot in red line, and it can be seen that strong magnetic disturbance occurred periodically. The proposed method calculated the weight of the IMU algorithm according to magnetic disturbance as described in Equations (15)–(17).

**Figure 11:**
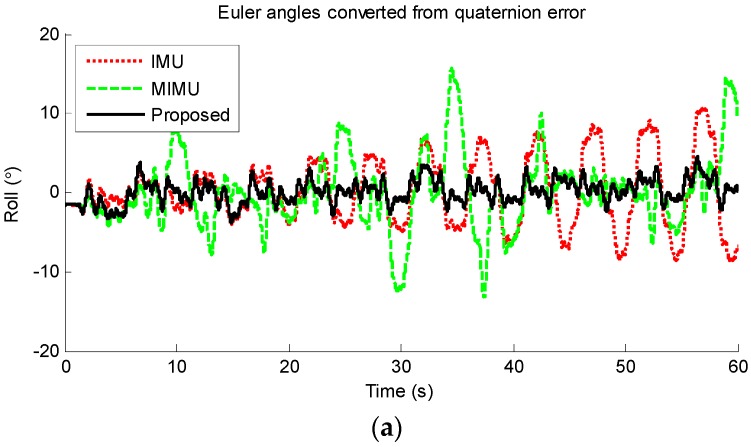

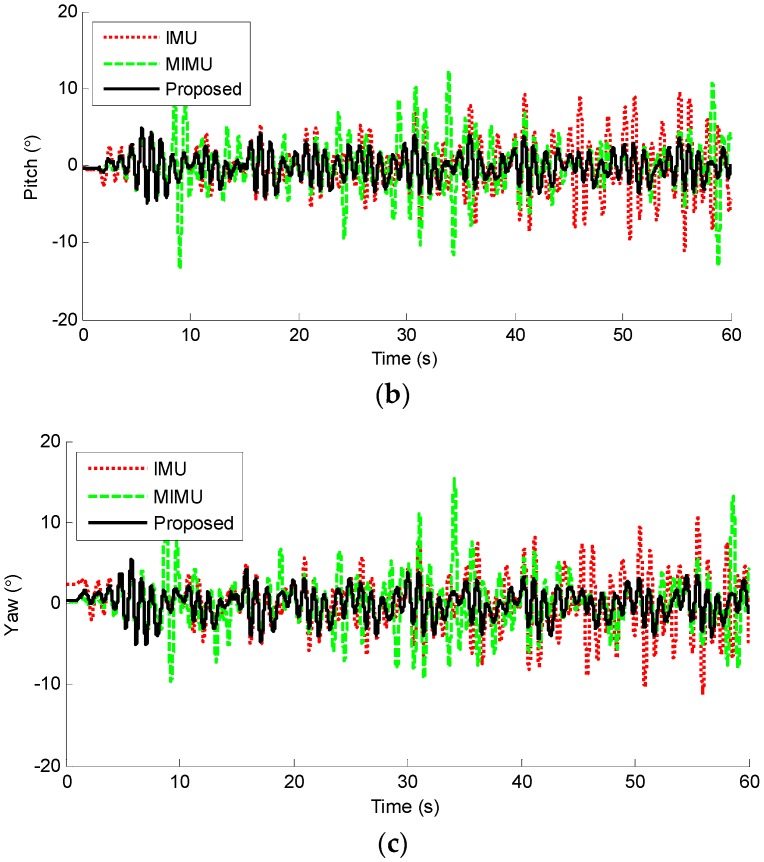
Euler angles converted from quaternion error among IMU algorithm, MIMU algorithm and proposed method from one example trial. (**a**) Roll angle; (**b**) Pitch angle; (**c**) Yaw angle.

**Figure 12:**
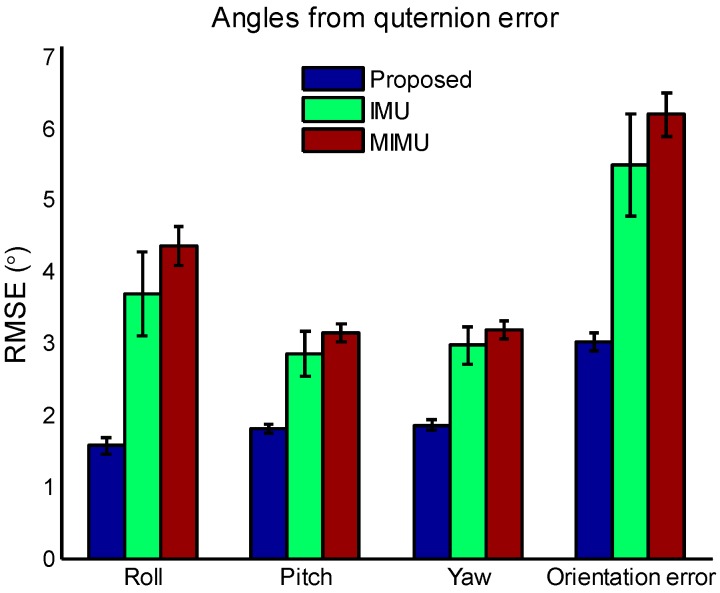
The mean and standard deviation of RMSEs of Euler angles and orientation error of all the ten trials. The bars represent the 95% confidence intervals.

Figure 11 shows the Euler angles converted from quaternion error among IMU algorithm, MIMU algorithm and the proposed method. Although these angles are no longer related to earth frame, the error trends are clear. It can be seen from Figure 11a that the error of IMU algorithm increases gradually, which indicates that IMU algorithm is suffered from drift. The MIMU algorithm is seriously impacted by the magnetic disturbance. Taking pitch angle error as an example, the error increased to more than 10° sometimes. While for the proposed method, the error is relatively small and stable. Thus, the detrimental effect of magnetic disturbance has been suppressed significantly. The mean and standard deviation of the RMSEs of all the ten trials are calculated and plotted in Figure 12. It can be seen that the error of the proposed method is smaller than original MIMU and IMU fusion algorithm, which demonstrates that the special handling for magnetic disturbances in the proposed method is effective. The mean orientation error of the proposed, MIMU algorithm and IMU algorithm are 3.02°, 6.19° and 5.48° respectively, and hence the orientation error of the proposed method is reduced by 51.2% compared with original MIMU algorithm. 

## 5. Discussion and Conclusions

Orientation estimation using magnetic and inertial measurement unit is an active research topic in the field of human motion analysis, and many sensor fusion algorithms have been proposed. Interestingly, the discrepancy of the accuracy of several state of art fusion algorithms is very small in magnetic disturbances free environment [8,13]. Therefore, the trend is not to proposed a new algorithm outperforming previous algorithms, but to place efforts on dealing with special situations, such as magnetic disturbances [22], high speed motion [33] and longtime monitoring [34]. Focusing on improving the estimated accuracy in the presence of magnetic disturbances, this paper proposes a novel adaptive method based on existing fusion algorithms. It is a combination of MIMU algorithm and IMU algorithm, so that can take the advantages of both algorithms.

For the sensor fusion algorithms based on MIMUs, accelerometer and magnetometer data are used for compensating the drift error of angular velocity integration. Compared with gravity acceleration, the geomagnetic field is weaker and more likely to be distorted by ferromagnetic materials in our surroundings. This problem is hard to overcome. Until now, many studies still take the orientation from optical motion capture systems rather than that from MIMUs as a reference orientation [32,35]. A considerable amount of literature focuses on mitigating the negative effect of the magnetic disturbances [16,17,18,19,21,22,36]. To the authors’ knowledge, the stationary state detection in the proposed method is a novel handling, which can be immune to any kind of magnetic disturbances in stationary state, even when the disturbance lasts for a long time. This feature is quite suitable for intermittent motion monitoring in daily activities. If a MIMU mounted on a human segment were moved near to ferromagnetic materials and kept still for some time, the yaw angle error would not change gradually.

In dynamic state, the proposed magnetic disturbance severity determination can be regarded as an improved method based on threshold-based method [22]. The magnetic disturbance weight λ in Equation (17) is continuously variable with external magnetic disturbances, which ensures the orientation updated smoothly without abrupt changes. And because the performance of the threshold-based method depends on its implementation and tuned parameters, it is difficult to get a fair comparison between threshold-based method and the proposed method through experiments. Comparing with the results in the literature, the proposed method can achieve equivalent accuracy versus the fine-tuned threshold-based method but with easier tuning process, and the achieved 51.2% error reduction is equivalent to 50–60% reduction in a fine-tuned method discussed in [24]. 

Moreover, another important feature is that the proposed adaptive method is a general method independent of fusion algorithms, which can be viewed as an adaptive combination of IMU and MIMU algorithms. Most of the current fusion algorithms contain IMU and MIMU versions [12,13,20]. Consequently, the proposed method can be easily implemented in various state of art fusion algorithms, appending the anti-magnetic disturbances feature to the main algorithms. In order to validate the proposed method, three sets of experiments were conducted on an instrumented gimbal, i.e., stationary state experiments, dynamic state with and without magnetic disturbance experiments. The estimation error of the proposed method was compared with those of the original IMU and MIMU gradient descent algorithms. Results show that the proposed method is immune to magnetic disturbances in stationary state, and achieves significant reduction in the error caused by magnetic disturbances in dynamic state. 

In our study, the experiments were performed on the instrumented gimbal. Although we tried to make the controlled condition similar to the actual applications such as the gait task experiments presented in [22,32], the performance is only representative for these controlled experimental conditions. Further validation with human subject experiments is required for further validating the proposed method. Compared with the gimbal experiments, human subject experimentation offers two challenges. First, the controllability and repeatability of human subject experiments are not as well as the instrumented gimbal experiment. Second, to evaluate the proposed method under real living environment, human subject experimentation on segment orientation estimations is still being planned.

In conclusion, this study presents a new adaptive method for dealing with magnetic disturbances, and the main contributions of this paper are listed below: First, we presented a preprocessing method including stationary state detection and the magnetic disturbance severity determination. Second, the proposed method can be viewed as an independent method to achieve adaptive combination of IMU and MIMU algorithms.
